# Emerging Standards and the Hybrid Model for Organizing Scientific Events During and After the COVID-19 Pandemic

**DOI:** 10.1017/dmp.2020.406

**Published:** 2020-10-26

**Authors:** Sara Hanaei, Amirhossein Takian, Reza Majdzadeh, Christopher Ryan Maboloc, Igor Grossmann, Orlando Gomes, Milos Milosevic, Manoj Gupta, Alireza A. Shamshirsaz, Amine Harbi, Amer M. Burhan, Lucina Q. Uddin, Arutha Kulasinghe, Chi-Ming Lam, Seeram Ramakrishna, Abass Alavi, Jan L Nouwen, Tommaso Dorigo, Michael Schreiber, Ajith Abraham, Natalya Shelkovaya, Wojtek Krysztofiak, Majid Ebrahimi Warkiani, Frank Sellke, Shuji Ogino, Francisco J. Barba, Serge Brand, Clara Vasconcelos, Deepak B. Salunke, Nima Rezaei

**Affiliations:** 1 Universal Scientific Education and Research Network (USERN); 2 Research Center for Immunodeficiencies, Children’s Medical Center, Tehran University of Medical Sciences, Tehran, Iran; 3 Department of Global Health & Public Policy, School of Public Health, Tehran University of Medical Sciences, Tehran, Iran; 4 Health Equity Research Center (HERC), Tehran University of Medical Sciences, Tehran, Iran; 5 Department of Health Management & Economics, School of Public Health, Tehran University of Medical Sciences, Tehran, Iran; 6 Community-Based Participatory Research-Center, Tehran University of Medical Sciences, Tehran, Iran; 7 Knowledge Utilization Research Center, Tehran University of Medical Sciences, Tehran, Iran; 8 Department of Epidemiology and Biostatistics, School of Public Health, Tehran University of Medical Sciences, Tehran, Iran; 9 Department of Philosophy, Ateneo de Davao University, Davao City, Philippines; 10 Department of Psychology, University of Waterloo, Canada; 11 Lisbon Accounting and Business School, Lisbon Polytechnic Institute (ISCAL-IPL), Lisbon, Portugal; 12 CEFAGE - ISCAL/IPL Research Center, Évora, Portugal; 13 Faculty of Physical Education and Sports Management, Singidunum University, Belgrade, Serbia; 14 Department of Mechanical Engineering, National University of Singapore, Singapore; 15 Division of Fetal Therapy and Surgery, Department of Obstetrics and Gynecology, Baylor College of Medicine and Texas Children’s Hospital, Houston, TX, USA; 16 Faculty of Social Sciences and Humanities, University of Souk Ahras, Algeria; 17 Department of Psychiatry, Schulich School of Medicine & Dentistry, Western University, London, ON, Canada; 18 Department of Psychology, University of Miami, Coral Gables, FL, USA; 19 School of Biomedical Sciences, Institute of Health and Biomedical Innovation, Queensland University of Technology, Brisbane, Queensland, Australia; 20 Translational Research Institute, Brisbane, Queensland, Australia; 21 Department of International Education, Education University of Hong Kong, Hong Kong, China; 22 Center for Nanotechnology, National University of Singapore, Singapore; 23 Department of Radiology, Hospital of the University of Pennsylvania, Philadelphia, PA, USA; 24 Department of Internal Medicine and Infectious Diseases, Erasmus Medical Center, Erasmus University, Rotterdam, Netherlands; 25 Istituto Nazionale di Fisica Nucleare, Sezione di Padova, Italy; 26 Institute of Physics, Technische Universität Chemnitz, Chemnitz, Germany; 27 Machine Intelligence Research Labs (MIR Labs), Auburn, WA, USA; 28 Department of Philosophy, Cultural Studies and Information Activity, Volodymyr Dahl East Ukrainian National University, Severodonetsk, Ukraine; 29 Institute of Philosophy and Cognitive Science, University of Szczecin, Poland; 30 School of Biomedical Engineering, University of Technology Sydney, Sydney, Australia; 31 Department of Cardiothoracic Surgery, Alpert Medical School of Brown University, Providence, RI, USA; 32 Department of Pathology, Brigham & Women’s Hospital (BWH), Harvard Medical School, Boston, MA, USA; 33 Department of Epidemiology, Harvard T.H. Chan School of Public Health, Boston, MA, USA; 34 Broad Institute of MIT and Harvard, Cambridge, MA, USA; 35 Nutrition and Food Science Area, Department of Preventive Medicine and Public Health, Food Science, Toxicology and Forensic Medicine, Faculty of Pharmacy, Universitat de València, València, Spain; 36 University of Basel, Psychiatric Clinics (UPK), Center of Affective, Stress and Sleep Disorders (ZASS), Basel, Switzerland; 37 University of Basel, Faculty of Medicine, Department of Sport, Exercise and Health, Division of Sport Science and Psychosocial Health, Basel, Switzerland; 38 Kermanshah University of Medical Sciences, Kermanshah, Substance Abuse Prevention Research Center and Sleep Disorders Research Center, Kermanshah, Iran; 39 Tehran University of Medical Sciences, School of Medicine, Tehran, Iran; 40 Departamento de Geociências, Ambiente e Ordenamento do Território/Unidade de Ensino das Ciências, Faculdade de Ciências, Universidade do Porto, Porto, Portugal; 41 Department of Chemistry & Centre for Advanced Studies in Chemistry, Panjab University, Chandigarh, India; 42 National Interdisciplinary Centre of Vaccine, Immunotherapeutics and Antimicrobials, Panjab University, Chandigarh, India; 43 Department of Immunology, School of Medicine, Tehran University of Medical Sciences, Tehran, Iran

**Keywords:** congress, COVID-19, event, pandemic, resilience, standard

## Abstract

Since the beginning of 2020, the coronavirus disease (COVID-19) pandemic has dramatically influenced almost every aspect of human life. Activities requiring human gatherings have either been postponed, canceled, or held completely virtually. To supplement lack of in-person contact, people have increasingly turned to virtual settings online, advantages of which include increased inclusivity and accessibility and a reduced carbon footprint. However, emerging online technologies cannot fully replace in-person scientific events. In-person meetings are not susceptible to poor Internet connectivity problems, and they provide novel opportunities for socialization, creating new collaborations and sharing ideas. To continue such activities, a hybrid model for scientific events could be a solution offering both in-person and virtual components. While participants can freely choose the mode of their participation, virtual meetings would most benefit those who cannot attend in-person due to the limitations. In-person portions of meetings should be organized with full consideration of prevention and safety strategies, including risk assessment and mitigation, venue and environmental sanitation, participant protection and disease prevention, and promoting the hybrid model. This new way of interaction between scholars can be considered as a part of a resilience system, which was neglected previously and should become a part of routine practice in the scientific community.

Progressively spreading throughout the world, the 2019 coronavirus disease (COVID-19) is affecting more and more individuals every day. Despite implementing various policies in the service of controlling the crisis by different jurisdictions, many countries initially underestimated the extent of this pandemic, while a few countries are still far from ideal control by mid-2020.^1-3^ Some of the reasons for the current global crisis include commercial globalization and consumer culture.^4^

Accordingly, as the importance of scientific events in promotion of knowledge transfer and scientific collaborations, this review was conducted to describe the current status of scientific events in the COVID-19 pandemic and propose a solution for overcoming the barriers. So, the scientific world can take 1 step forward toward organizing efficient and safe scientific events in the COVID-19 pandemic.

## Limitations Due to COVID-19

To prevent the spread of severe acute respiratory syndrome coronavirus 2 (SARS-CoV-2), human gatherings of any kind have been limited and replaced with alternative options, such as remote working, virtual or hybrid education and conferences, and cancellation of many in-person social or religious events, as well as overseas travel.^5^ Governments decided to set more limits and borders for people, while the virus knows no border.^6,7^ Although such restrictive policies might be the only viable option in the short-run, they are not cost-effective, efficient, and even feasible in the mid- and long-term scenarios for many businesses and countries in cases of prolonged global disasters, such as the COVID-19 pandemic, especially in low- and middle-income countries. Emergencies such as the COVID-19 pandemic happen, while citizens’ financial, educational, scientific, emotional, and cultural needs must be adequately fulfilled. This paradoxical situation of addressing both the emergency and people’s needs might be worse in the settings with underlying conditions that suffer the dual burden of pandemic and other threatening situations.^8^

## Pandemics are Here to Stay

Pandemics and their unintended consequences are expected to stay with human societies for an undefined time to come. Pandemic-related challenges have affected almost all aspects of our lives and continue defining new standards for a “new normal.” This new normal is evolving, as the pandemic progresses to match with the complexities of modern life. According to Jerome Ravetz,^9^ the term *new normal* is not adequate to capture the complexity of the COVID-19 pandemic. Given the constant evolution of what the “new normal” may look like, it may be preferable to talk about resilience in the face of the emerging standards, complemented by an extended awareness of evolving problems and solutions.

## Shifting Event Organization From In-Person to Virtual

Similar to many global gatherings (eg, the Olympics, the Haj pilgrimage), scientific events, including conferences and symposiums, have been heavily affected by this worldwide pandemic. Ever since the World Health Organization (WHO) declared the pandemic on March 11, 2020,^10^ almost all scheduled scientific events (including global congresses, symposiums, conferences) for most scientific societies have either been postponed, canceled, or held virtually. As the most important global political decision-making gathering for health, the WHO, for the first time in its history of over 70 years, held the 73rd annual World Health Assembly (WHA) virtually and was much shorter than usual, in May 2020.^11^ Managing important events during a pandemic is not just about how to deal with restrictions in the area of planning and coordination. It will also require active democratic participation from all stakeholders. The extent and pace of the virtualization adoption rate have been faster since the start of the pandemic.

## Advantages and Disadvantages of Virtual Events

Advantages of virtual events include increased inclusivity and accessibility and reduction of the carbon footprint. At this moment, however, virtual meetings are not as effective as in-person events, in part, due to several reasons as follows:

1. *Socialization and networking*: Humans are social beings, and the social dimension comprises an important aspect of our lives. As a social phenomenon, many human beings are yet uncomfortable to replace the feeling behind face-to-face or whole-body communications with virtual conversations. For example, social greetings are more meaningful in face-to-face meetings, where eye contact and body language can be better interpreted.^12^ As one of the most fundamental byproducts of physical gatherings, socialization and networking could be considerably compromised in virtual events. It would not be exaggerating to say that establishing new scientific collaboration is the main reason for many conference attendees, which is more feasible through in-person gatherings. For instance, participants post their questions in a chat-box and subsequently a host reads those questions to the speaker. This way, virtual meetings provide some means for limited scientific communication. At the same time, the opportunities for academic exchange and discussion that typically take place in the hallways of scientific events, during tea and lunch breaks, are missed. You can add tourism and social tours to the advantages list, which are more attractive when one can walk through and experience the novelty of a new country or city. Such social events provide opportunities for sharing positive social experiences likely fostering connections and team building. Overall, in-personal intercultural encounters and cultural exchanges allow for better understanding the nuances of different cultures and their approaches to collaborative research.

2. *Internet interruptions due to the poor or limited connectivity*: Not every part of the world has (equal) access to high quality Internet services. Consequently, virtual conferences can easily get interrupted, if one of the connecting parties has poor Internet. Bandwidth-based disruptions can prevent achieving the scientific goals of a congress, and bringing together global leaders across the world may be challenging. Adopting to new platforms of virtual connections requires learning and following new online etiquette.^13^ At times, participants may forget to mute their audio, leading to unnecessary disturbance to the speaker. Furthermore, poor connection or electronic glitches in some places can lead to participants raising their hand virtually to indicate that something is wrong, in general, with the whole system. Such technical challenges and other emerging issues are typically absent in face-to-face meetings.

3. *Formality and efficiency*: A virtual model for scientific gatherings can reduce registration and travel costs, and reduce the carbon footprint. At the same time, in-person meetings allow more formal engagement of participants and more fluent exchange of ideas and practical points within the well-established long tradition of conference processes that allow interactivity, dialogue, and open scientific debate due to a visibly diverse background of researchers.^14^

4. *The pace of producing new ideas*: Virtual meetings provide an opportunity for equal contribution. The implicit criteria for presenting in virtual meetings during the COVID-19 era should be meticulously studied, given that some people in each scientific field are usually more dominant and have more chances of giving talks in several webinars at a time. They might be better communicators, have a high level of language proficiency, oversee important positions, or attract more viewers resulting in a possible danger of providing more voice to these individuals as compared to other intelligent, but potentially less known contributors to the scientific discourse. Webinars are usually conducted in a short time period and may not provide enough time or modalities to criticize talks. This reduces the chance of exposure to a counter idea through virtual meetings. Therefore, there is a chance of being led by a presenter without supporting evidence because they are presenting in more virtual sessions. However, the spread of ideas and increasing pace of information flow do not guarantee their quality. A wise handling of information requires careful deliberation and an open debate of presented ideas^15-17^ – something that may be more effectively implemented in-person as opposed to online.

## Potential Solutions for Scientific Events

For the abovementioned reasons, in-person meetings cannot be fully replaced with virtual ones. Robotic teachers are less preferred by university students than human teachers. Another example of this may be, when e-books were first introduced to the world, many were excited to test them and even replace printed books with them. Nevertheless, printed books have their unique appeal and many people still prefer paging through printed text books rather than reading on an electronic device.^18^ In turn, publishers who offer both printed and electronic versions are usually more successful, since they respond to the needs of both groups of users: e-book readers and printed book readers. Similarly, the hybrid model with both virtual and in-person participation might be a sensible approach to organize scientific events during the emerging normal routines of the COVID-19 pandemic.

## Emerging Standards for The Hybrid Model

**Figure 1.**
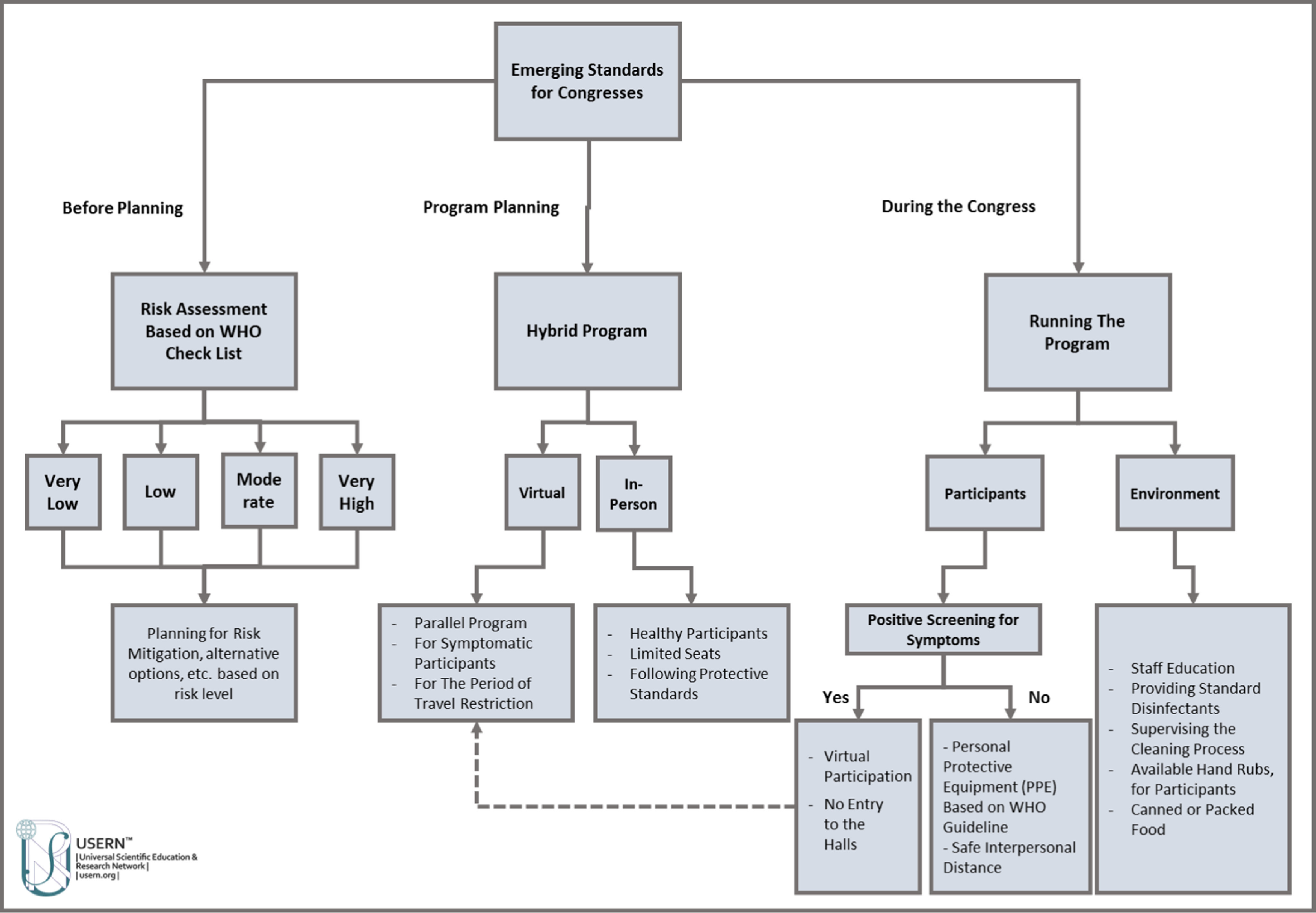
Defining hygienic standards for organizing congresses during and after the COVID-19 pandemic.

As the world is adopting emerging normal protocols for various gatherings, including scientific events, defining new sanitary standards is an essential step toward the emerging normal condition in organizing scientific meetings and congresses. The WHO has provided various sets of guidelines with regard to infection prevention and control in different situations (eg, workplaces, schools, mass/religious gatherings, sport events).^19^ Such guidelines could be modified for their specific use in organizing congresses/conferences as well. We propose a fourfold strategy, as shown in Figure 1.

1. *Risk assessment and mitigation*: Perform risk assessment before planning for a congress in the context of the COVID-19 pandemic, to consciously judge the probability of infection transmission and make the most rational decisions to mitigate the possible governing factors.^20^ WHO guidelines suggest to gather the following meeting information: date and place (country, city) of meeting, number of participants, proportion of high-risk groups, international participation (number of foreign participants, their countries of origin, and their COVID-19 status), restriction status and health prevention measures in the host country, social events, and preventive methods in the congress. Score and categorize the risks as very low, low, moderate, or very high levels, and plan for mitigation of risks accordingly.^20^

Before the coronavirus pandemic, people understood that the probability of risk is crucial before one can make any logical decision about an activity. When the risk is low, then the rational thing to do is to proceed with a plan in mind. However, when it comes to the issue of safety, the idea of hazard is the most relevant element to consider. In a post-pandemic world, safety must always be the topmost priority.^9^

2. *Venue and environmental sanitation*: As COVID-19 can remain on and be transmitted through the environment and surfaces, disinfect and sanitize the congress venues to prevent disease spread. According to the WHO guidelines,^21^ all staff and executive members in charge of venue preparations should be properly trained about the cleaning methods and use of efficient disinfectants. The ingredients of disinfectant products should meet the standards to efficiently clean all surfaces to reduce the risk to the participants and event organization personnel.^21^ Dedicated personnel should provide careful health and safety monitoring and supervise the disinfecting processes. It is essential to make hand sanitizers widely accessible for participants at different locations of the venue. In addition, institute physical distancing rules when arranging the seats in the conference halls, and, for social interactions in session breaks, mandate at least 1.5 meters between the seats/participants interacting. Finally, provide all lunches and snacks in eco-friendly, disposable packages, and avoid buffets.

3. *Participants protection and disease prevention*: WHO guidelines for appropriate personal protective equipment suggest to pay attention to specific needs of each group (eg, high-risk groups with comorbidities, > 65 years of age, children).^22^ For the sake of disease prevention and control, no participants with symptoms of COVID-19 infection should be allowed to attend the conference. Also, according to the Centers for Disease Control and Prevention (CDC), any individual who had close contacts with COVID-19 patients in the previous 14 days should be quarantined and monitored for symptoms. A close contact is defined if any of the following items presents: (a) having at least 15 minutes of contact with a COVID-19 patient from less than 6 feet of distance, (b) taking care of a COVID-19 patient at home, including first-degree family members, (c) direct physical contact with a COVID-19 patient, (d) contact with respiratory droplets of a COVID-19 patient through cough or sneeze.^23^ In this context, complying with the social duty plays a crucial role in breaking the disease transmission chain, since these asymptomatic individuals could be disease carriers.

At the time of entry, each participant should provide a recent negative COVID-19 test result and be screened based on symptoms and body temperature. In case of any suspicion, the delegate will not be allowed to attend the in-person congress. Equip all participants with personal protections (especially surgical face masks), and ensure access to the appropriate amount of hand sanitizers during the congress. Ensure to comply and examine seating arrangements throughout the congress. All participants ought to comply with keeping safe interpersonal distance during their communications with others and provide contact details during the congress for potential contact tracing.

4. *The hybrid model*: Planning a hybrid model and having parallel virtual sessions could be advantageous not only for symptomatic participants, but also for international speakers/participants who cannot attend the congress in-person due to travel and quarantine restrictions that may be in place at the conference location by the host country. At the same time, such restrictions will not be applicable to local participants and they may benefit immensely by physically attending such events, while they can choose whether to attend virtually or in-person. This hybrid model could take the form of local in-person hubs, including a small number of participants, in parallel with online and virtual activities, including lectures that are available to wider audiences. Although social interactions are more efficient in the in-person gatherings, virtual participants can benefit from social interactions through a social portal, where speakers can virtually log in and have discussions with in-person and virtual speakers.

In parallel with the adjustment of the standards of live events, it is necessary to further improve the organization of the virtual part of events. When the borders are closed and air traffic is interrupted, virtual events remain the only viable solution. To avoid and prevent some of the abovementioned shortcomings of virtual scientific events, it is necessary to move toward the medium of virtual reality (VR). There are many benefits that VR offers, from simulation of the presence of the event itself to the simulation of coffee breaks and informal parts of the event in which it is possible to engage in more intensive communication “face-to-face.” Moreover, there is a need of a highly specialized app to manage the lectures and events, so as to improve the speakers and listeners experience during the event. These apps will also help connect the researchers, which is one of the important aspects of scientific events. On the other hand, although it may seem that this type of event organization requires extensive complicated technology, in fact, it is possible today to consume content in VR with the help of a smartphone and cardboard or plastic glasses. The biggest obstacle still is the quality of the Internet connection, which research institutions should provide to their employees.

## Conclusion

As the COVID-19 (SARS-CoV-2) pandemic has demonstrated, science and knowledge sharing are vital to face global disruptive challenges. The post-normal scenario after COVID-19 must consider the societal aspects of every scientific event or gathering. They are actually as important as the biological concerns of people.^9^ Scientific gatherings have an important role in promoting science and network expansion for greater collaboration. Perhaps the most important aspect of such events is bridging the communication gap between scientists and researchers across the planet, which will enable the scientific progress. Scientists all over the world are capable not only of maintaining the already existing networks, but also engaging in new collaborations. These new collaborations are essential, mainly because they promote contact across disciplines. The problem we are facing is pervasive and widespread, demanding a transdisciplinary response.^24^ A recent OECD (Organisation for Economic Co-operation and Development) report points out the need to adopt a transdisciplinary attitude toward scientific research, given the complex nature of the societal challenges we are facing.^25^

Currently, the world is witnessing one of the greatest challenges to socioeconomic development in recent history. While scientific gatherings are still crucial to maintain, even in the time of crisis such as pandemics, convening these events needs to be continued in line with protective standards. Plans of emerging standardizing are crucial for a hybrid program, which allows both in-person and virtual participation. Virtual communication might be useful, but it cannot adequately replace in-person communication for much of body language, which people from many high-context cultures (eg, Arab countries, China, Russia, Japan) rely on.^26,27^ Without accounting for aspects of the body language and the social context, many aspects of interpersonal interaction may be lost in translation. The capacity of response to a shock is called *resilience*. The COVID-19 crisis showed that many systems, for example, financial and health systems, need resilience to avoid disruption and collapse due to unforeseen circumstances. COVID-19 presents a significant threat in our lives, but, without any doubt, it will not be the last shock of this kind. As with many other social systems, the scientific community needs a resilient way of communication. It is worth ending on this note: This will be neither the last human crisis nor the last pandemic in our lifetime. Consequently, it is crucial to maximize our learning curve now to prepare for upcoming pandemics.
